# Open‐Source Marine Biodiversity Data Quality in the Norwegian Sea Spanning 149 Years: Knowledge Gaps in the Deep‐Sea Mining Opening Area

**DOI:** 10.1002/ece3.71852

**Published:** 2025-08-26

**Authors:** Laura C. Paiba‐García, Geir Johnsen, Sam Wenaas Perrin, Torkild Bakken

**Affiliations:** ^1^ Department of Biology Norwegian University of Science and Technology Trondheim Norway; ^2^ University Centre in Svalbard Department of Biology Longyearbyen Norway; ^3^ Gjærevoll Centre for Biodiversity Foresight Analyses Norwegian University of Science and Technology Trondheim Norway; ^4^ Department of Natural History NTNU University Museum, Norwegian University of Science and Technology Trondheim Norway

**Keywords:** deep‐sea mining, environmental management, marine species pool, Norwegian extended continental shelf, open‐access biodiversity data, species occurrences, species richness

## Abstract

This work identifies spatial–temporal patterns of marine species biodiversity in the Norwegian, Greenland and Barents Seas and provides specific information in Norway for Environmental Impact Assessments and Statements about area‐based indices for biodiversity. The opening of the Norwegian Extended Continental Shelf for deep‐sea mining is a currently relevant topic for environmental management, as strategies to minimize mining impacts and delimit key zones for ecological preservation have been widely advised. A quality control procedure covering temporal and spatial scales on open‐source biodiversity data was applied, including the compilation of marine species from the archives of the Norwegian North‐Atlantic Expedition 1876–1878. Here, we present biodiversity patterns for 10,505,496 marine occurrences from 1876 to 2025 (149 years). Data occurrences were classified into two main datasets (shallow, < 500 m and deep ≥ 500 m) and two sub‐datasets for each (planktonic and benthic). 97% of the total were classified in the first main and 3% in the second main. On map view and out of 122,955 grid cells, 32,274 and 15,528 encompass data from the shallow and deep datasets, respectively, with different degrees of coverage inside; most frequently, grid cells with 1 to 10 occurrences. Data is mainly planktonic (20,098 grid cells for shallow‐planktonic and 3127 grid cells for deep‐planktonic). Peaks of species richness occur from southern to northern latitudes, even with evidently reduced values for species occurrences and abundances at certain latitudes. We conclude that knowledge gaps of benthic biodiversity in the Norwegian deep‐sea mining opening area are huge. The cumulative curve of species richness reveals that species identities, included in deep‐sea data, are not sufficient to quantify area‐based biodiversity indices in the species pool. Our findings are congruent with the need to contemplate data from deeper areas for decision‐making at different spatial–temporal windows, especially considering the granting of deep‐sea mining licenses.

## Graphical abstract

To identify spatial–temporal patterns of marine species biodiversity and to provide specific information in Norway for Environmental Impact Assessments and Statements, we present biodiversity patterns for marine occurrences from 1876 to 2025 (149 years). The knowledge gaps of benthic biodiversity in the Norwegian deep‐sea mining opening area are huge, and species identities included in deep‐sea data are not sufficient to quantify area‐based biodiversity indices in the species pool.

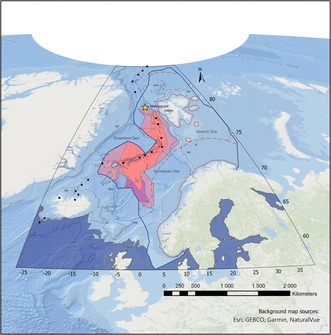

## 1 Introduction

The Norwegian extended continental shelf has been subject to recent attention for deep‐sea mining activities approved by the Norwegian parliament (das Neves 2024), covering areas of the Norwegian and Greenland Seas. Under the premise that a zero net loss of biodiversity is an impossible goal for deep‐sea mining (Niner et al. 2018), a hierarchy of mitigation measures has been proposed (Durden et al. 2017). The first measures correspond to avoidance and minimization of mining impacts on the surrounding ecosystem, such as removal of habitat substrate and organisms, disturbance by plumes of sedimentation and presence of pollutants and toxic molecules in distinct temporal and spatial scales (Hauton et al. 2017; Orcutt et al. 2020; Van Dover et al. 2017; Washburn et al. 2019; Williams et al. 2022), especially in areas with hydrothermal vents (Niner et al. 2018; Van Dover 2011; Van Dover et al. 2017). Currently, there are knowledge gaps preventing accurate characterization of pelagic and benthic communities, especially regarding polymetallic nodules, seafloor sulfides (i.e., polymetallic sulfides, produced at hydrothermal vents) and cobalt‐rich ferromanganese crusts (Amon et al. 2022; Jones et al. 2021; O'Hara et al. 2020; Ramirez‐Llodra et al. 2020). This is a relevant matter, as those mining processes have been compared to the scale of open‐cut mining and may occur in a widely unexplored ecosystem (Van Dover 2011).

Marine areas need to rely on the assessment of potential impacts of deep‐sea mining when addressing knowledge gaps. The deep sea is a complex mixture of environmental abiotic (e.g., sedimentation, light, temperature, salinity, nutrients, current speed and direction, and O_2_ and CO_2_ concentration) and biological variables (competition, prey–predator relationship and different substrate preferences) affecting biodiversity, function, health state and survival (Johnsen et al. 2020). This ecosystem is a zone devoid of photosynthesis and photosynthetic organisms, and light intensities are far below the theoretical minimum for the process (Johnsen et al. 2021). This applies to chemosynthetic biological communities and highly specialized fauna, such as those distributed along the Arctic Mid‐Ocean Ridge (AMOR) (e.g., Loki's Castle vent field), whose patches are highly vulnerable from mining impact (Eilertsen et al. 2024). These habitats are related to sulfur‐rich substrates (Ramirez‐Llodra et al. 2020; Tunnicliffe 1991), an ore of interest due to its content of metals of commercial value (Dumke et al. 2018). Knowledge gaps occur parallel to the granting of exploration licenses for deep‐sea mining by the International Seabed Authority (ISA) in Areas Beyond National Jurisdiction (ABNJ) (Amon et al. 2022; ISA 2022). This highlights the importance of setting in place robust regulatory frameworks for deep sea mining activities (e.g., Howard et al. 2020) before exploitation phases of mining can take place (Amon et al. 2022) and during mining phases of exploration.

To derive existing species data into management‐relevant information, it is important to consider how to link existing data with basic parameters (i.e., ecological indices) often used for biodiversity assessments. Ecologically, to relate the regional influence of species present in a given area to the community structure of a given locality, the concept of the species pool is used (Cornell and Harrison 2014). The structure in an ecosystem may be quantified by diversity indices and is related to ecosystem functions (Steinberg and Geller 1994; Fedor and Zvaríková 2019). A regional and a local scale are defined above and below 10 km^2^ respectively, with the second reaching orders of magnitude of 10^5^ and 10^6^ km^2^, coincidental with biogeographic boundaries of the size of continents or islands (Caley and Schluter 1997; Chase et al. 2019; Shurin et al. 2000). Ecosystem functions may be transferred from a regional area to a local area through processes of dispersion and environmental barriers, building up a filtered pool which determines community diversity (Cornell and Harrison 2014). As species identities are known in the species pool, an asymptote is seen in the cumulative taxon sampling curve (i.e., accumulation curve), which derivates in statistical calculations (e.g., rarefaction curves) allowing the normalization of sampling efforts in a community (Gotelli and Colwell 2001). Rare species are especially important for quantification of the species pool (Cornell and Harrison 2014) as are newfound ones, shaping species accumulation curves (e.g., Dorazio et al. 2006). Remarkably, as new data may mean new species in the pool, latitudinal gradients that may occur in a wide regional area could be uncovered (e.g., Rex and Etter 2010).

The Norwegian government has prepared literature reviews pointing to considerable knowledge gaps. However, it is not clear if there is enough biodiversity data to calculate area‐based biodiversity indices (i.e., Shannon or Simpson), estimated under completeness of the species pool including the mining opening area. Globally, possibly the most extensively studied area for deep‐sea mining has been the Clarion‐Clipperton Zone, located in the Pacific Ocean, where it has been advised that ore‐rich areas should be included in the Areas of Particular Environmental Interest (APEI) (Washburn et al. 2021). For the Environmental Impact Assessments/Statements (EIA/EIS) reported to the International Seabed Authority (ISA) for mining exploration and to the present date (ISA 2025), the German and Belgian locations have been basis of knowledge to study these particular zones, for example. Additionally, there have been multiple sources citing the potential effects of deep‐sea mining and the relevance of characterizing hydrothermal systems that may be affected by the activity (e.g., Ramirez‐Llodra et al. 2020; van Doorn et al. 2022). Here we address knowledge gaps to aid decision‐making processes, applying a specific procedure for assessing data quality in the area of interest (AOI). The aim of this work is to use the wide spatial coverage and availability of open‐source biodiversity data to explore temporal and spatial patterns in the extended Norwegian Continental Shelf, applying quality control on biodiversity data spanning 149 years.

## 2 Materials and Methods

### 2.1 Area of Interest

**FIGURE 1:**
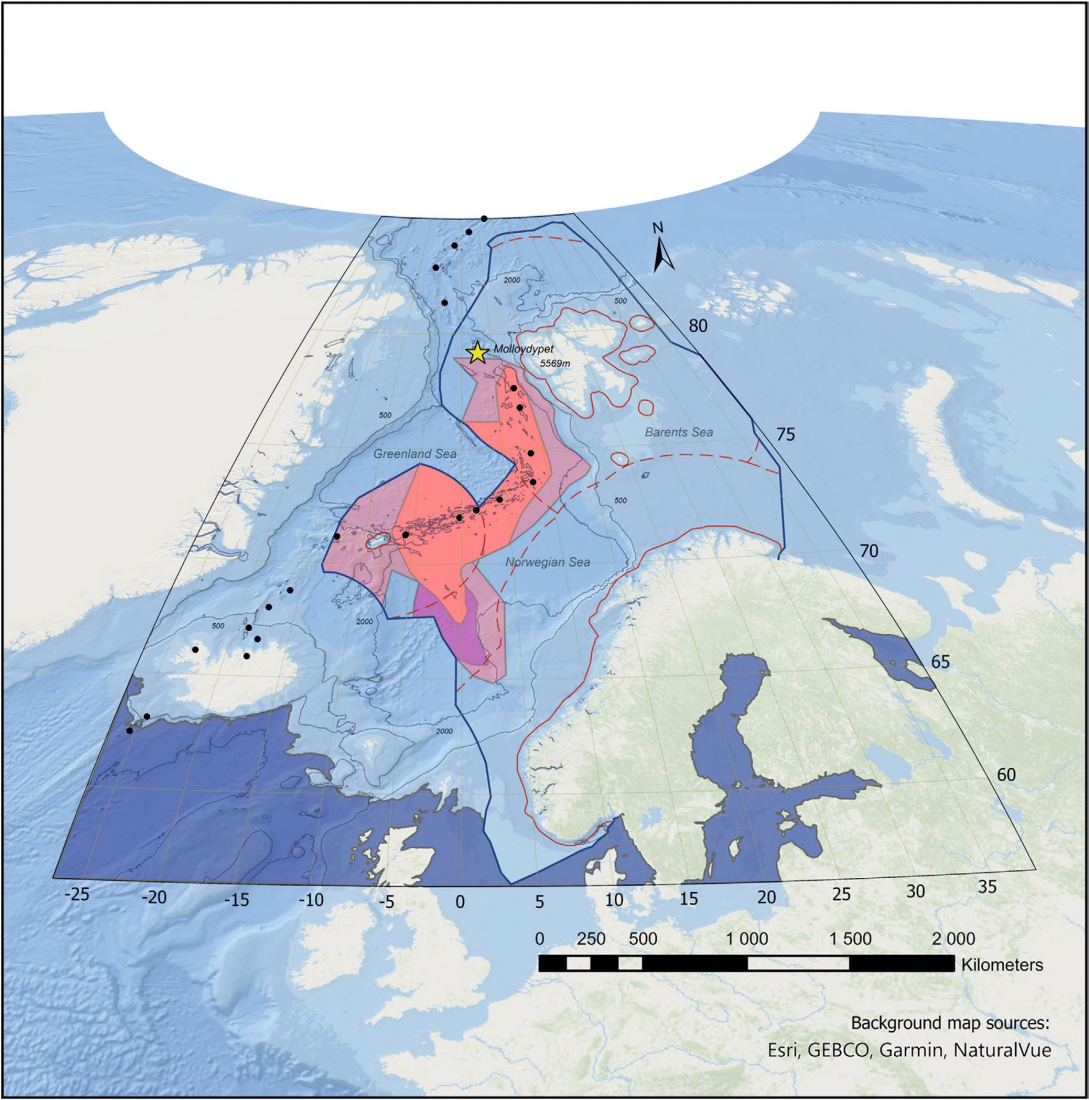
Area of Interest (AOI), Norwegian and Greenland Seas. Pale red (593,845 km^2^), bright violet (328,950 km^2^), and vivid red (281,208 km^2^) regions represent the assessment, proposed and opening areas for deep‐sea mining, respectively (NOD, 2024). The solid dark blue line is the boundary of the extended Norwegian Continental shelf, the dashed red line the boundary of the EEZ (Exclusive Economic Zone) and the solid red line the Norwegian Territorial Border (NOD, 2025). Note that the proposed area covers the opening area and the visible area in bright violet. Black dots represent the Hydrothermal Vents GIS data—InterRidge Global Database of Active Submarine Hydrothermal Vent Fields of the Nauru Environmental Data Portal (Nauru, 2025)‐ and the star locates the *Molloydypet* (5569 m of depth). Contour lines are the isolines for 500 m and 2000 m depth and were retrieved in December 2024 from the bathymetric contours of the EMODnet portal, available at https://emodnet.ec.europa.eu/geoviewer/. The map is displayed under the equal‐area projection of Aitoff (EPSG Geodetic Parameter Dataset: 54043) and latitude and longitude axes are plotted in decimal degrees. Imagery reproduced from the GEBCO Basemap (NOAA NCEI Visualization) in ArcGIS (Credits, map images: GEBCO Compilation Group 2024 and the cited background map sources).

The Area of Interest (AOI) is defined as the North Atlantic north of the Shetland‐Faroe‐Iceland‐Greenland ridge, and a part of the Polar Sea (Figure 1). The northern boundary is the Yermak Plateau (north of Svalbard) and the northern definition of the Norwegian continental shelf (85° N). The eastern limit is the Norwegian coast, including the easternmost definition of the Norwegian continental shelf in the Barents Sea (38° E). The southern boundary is the northern margin of the Iceland‐Faroe barrier and the southern definition of the Norwegian continental shelf (56° N). The western boundary corresponds to the limit between the basins north of the barrier and the north of the Irminger Sea (27° W). The AOI follows a design to encompass the assessment, proposed, and opening areas for deep‐sea mining in the Norwegian and Greenland Seas (Figure 1).

Boundaries between oceanic provinces and positioning of the continental shelf break are the criteria to choose how to handle data in the AOI. The area covered by the AOI includes all the oceanic provinces from the surface to the trench *Molloydypet*, which contains the deepest point in Norway (5569 m) (Dipper 2022; GEBCO Compilation Group 2024; Jacquemont et al. 2024; Kingsford 2024; Setså 2023; Zezina 1997). These provinces are the sublittoral/epipelagic (0–200 m), the sublittoral‐bathyal/mesopelagic (200–1000 m), the bathyal/bathypelagic (1000–4000 m) and the abyssal/abyssopelagic (4000–6000 m). While the boundary marking the start of the deep sea is usually designated as 200 m, the east boundary of the Norwegian Sea presents a pronounced continental shelf break diving to the west, especially along Norway and Svalbard, then coinciding closely with the 500 m depth isoline (Blindheim and Østerhus 2005). This marked change in bathymetry matches oceanographical regimes (Blindheim and Østerhus 2005) related to a considerable faunal shift, making 500 m a more appropriate boundary in the context of this work (Høisæter 2010; Oug et al. 2017). Additionally, the continental slope along the south of the Yermak plateau, the east of the Norwegian Sea, the north of the Shetland‐Faroe‐Iceland‐Greenland ridge, and the west of the Greenland Sea goes from 500 m to 1000 m in a distance of < 200 km, in a wide part of its extension (see the GEBCO bathymetrical chart of the oceans by the GEBCO Compilation Group 2024). Therefore, a first depth zone above 500 m is defined for this study and named the shallow dataset. Below, the whole marine volume is divided into equal 500‐m segments bathymetrically, summing to 11 depth zones here named the deep dataset, which covers the bathyal and abyssal benthic provinces offshore the continental shelf break.

### 2.2 Data Collection, Quality Assessment, and Data Analysis

**FIGURE 2:**
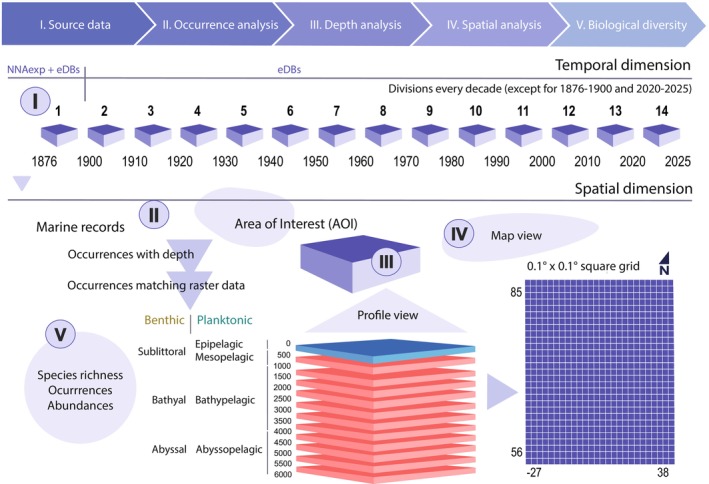
Quality control for biodiversity data in the AOI. The composed dataset was divided into 14 temporal segments, named as decades. Note that the electronic databases cover from 1876 to 2025 and the Norwegian expedition covers 1876–1878 specifically. NNAexp: Norwegian North Atlantic Expedition 1876–1878, eDBs: Electronic databases (GBIF, OBIS, and EMODnet). Occurrences matching raster data (*.tiff* bathymetric files, see depth analysis in materials and methods) refer to occurrences which had a raster pixel equivalent for their geographical position. Blue corresponds to the shallow dataset and red, to the deep dataset.

Biodiversity occurrence data from open‐source databases and paper archives was retrieved and filtered to obtain exclusively marine species occurrences (i.e., each row composing the databases), per decade, in the AOI. Quality assessment is described in Figure 2. Data analysis was run through R (R Core Team 2024) and a rectification for geographical positioning of shapefiles and data points was supported with ArcGIS. All relevant code can be found at the GitHub repository Marine‐biodiversity‐in‐the‐Norwegian‐deep‐sea‐mining‐opening‐area, following the roman numeral of the step for which scripts were designed.

#### 2.2.1 Source Data

Data was downloaded in April 2025 from the Global Biodiversity Information Facility (GBIF), the Ocean Biodiversity Information System (OBIS) and the European Marine Observation and Data Network (EMODnet), for all occurrence data from 1876 to the present time, through Application Programming Interfaces (API) (Table 1). These electronic databases were complemented with occurrences from the multi‐volume book series from the Norwegian North‐Atlantic Expedition 1876–1878 (Mohn et al. 1880). For the latter, sampling locality coordinates and species occurrences were digitized from the books and included for this study. All volumes of the expedition were revised, and species names were tabulated along with their station collection data and the depth at which they were found. First, selected electronic data columns are part of the Darwin Core standard (DwC, see https://dwc.tdwg.org/) and were related to four identifiable features: location features (i.e., *decimalLongitude, decimalLatitude, coordinateUncertaintyInMeters, coordinatePrecision*), depth features (i.e., *depth, depthAccuracy*), taxonomic features (i.e., *class, family, kingdom, scientificName, taxonRank, individualCount*), time features (i.e., *dateIdentified, day, eventDate, month, year)* and identification features (i.e., *basisOfRecord, catalogNumber, collectionCode,* *id, issue, flags, dataset*). Then, occurrences recorded with no value of depth were added with consistent values for their column *depth* (i.e., NA, “not available” values). Columns from the electronic databases were chosen to find an equivalent among them (e.g., *gbifID* in GBIF and *id* in OBIS). The WGS datum for data corresponds to WGS84 (EPSG Geodetic Parameter Dataset 4326), which was maintained through the process for geographical consistency. The corresponding scripts in GitHub are Ia to Id.

**TABLE 1:** Packages for API access (Application Programming Interfaces) to electronic databases. The keys for the downloaded GBIF sets are available in the GitHub repository.

Database	Date of access	API's package	Name	Source
GBIF	Apr‐25	*rgbif*	Interface to the Global Biodiversity Information Facility API	Chamberlain et al. (2024); Chamberlain and Boettiger (2017)
OBIS	Apr‐25	*robis*	Ocean Biodiversity Information System (OBIS) Client	Provoost and Bosch (2022)
EMODnet	Apr‐25	*EMODnetWFS*	EMODnet web service documentation	Krystalli et al. (2023)

#### 2.2.2 Occurrence Analysis

Source data was masked first using a .*shp* (shapefile) file of the south of the Shetland‐Faroe‐Iceland‐Greenland ridge. After, with a shapefile of the landforms from the command “*ne_countries*” (“*Get natural Earth world country polygons*”) from the package “*rnaturalearth*” (Massicotte and South 2023), terrestrial occurrences were excluded. Species level was filtered from data through the column “taxonRank” (from the DwC) and marine species names were compared to accepted and related‐to‐those accepted species names, through a World Register of Marine Species (WoRMS) database dump (WoRMS Editorial Board 2024). Therefore, non‐marine taxa were filtered out from further analysis. Additionally, one taxonomical validation list revised manually was used (“one”, available in GitHub) and valid names were added manually in the code (see the GitHub repository). The corresponding script in GitHub is II.

#### 2.2.3 Depth Analysis

**FIGURE 3:**
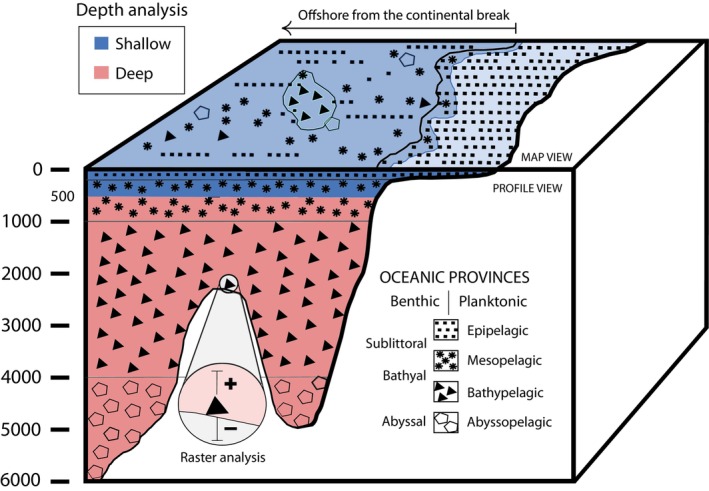
A schematized 3D view of the depth analysis (depth‐contouring and raster analyses). This analysis (1) seeked the identification of occurrences in the shallow (blue) and deep (red) datasets geographically, with emphasis in occurrences without depth information and (2) the identification of planktonic and benthic data. The symbols represent how the occurrences are shown in a profile (vertical) view compared to a map (horizontal) view. The shallow dataset is divided into the sublittoral/epipelagic area and the sublittoral/mesopelagic area. The black line between the shallow and deep datasets in map view marks the exemplification of the continental shelf break along the 500 m contour line. The red area represents the deep dataset. The + and − in raster analysis depict the range of depth accuracy for a given occurrence.

Depth analysis consisted of two analyses (Figure 3). The outcome of the analyses gave information about the oceanic provinces in which occurrences are located and resulted in producing two main datasets for species: shallow and deep (< 500 m and ≥ 500 m deep, respectively) and two sub‐datasets in each: planktonic and benthic, therefore four in total. As geographical positioning of the coastline between data from R and ArcGIS did not precisely coincide, datapoints that were not covered by any bathymetric contour and lay on the coast, were included in the shallow dataset. The land shape file provided by R has its source from the North American Cartographic Information Society, in the package *rnaturalearth* (“*World Map Data from Natural Earth*”), and bathymetric contours were built from the World Ocean Base by ArcGIS (Credits: Esri, GEBCO, Garmin and NaturalVue) with the tool “contour”. To locate occurrences geographically, depth polygons were designed with depth contours every 500 m in ArcGIS (datum WGS84, EPSG Geodetic Parameter Dataset 4326) and imported to R to perform the analysis. The scripts for the depth analysis are IIIa and IIIb.

The first analysis is called depth‐contouring analysis, where occurrences without depth were classified into either the shallow or deep dataset, and occurrences with depth were rectified geographically to confirm if they belonged to either of these sets or were possibly misplaced geographically (Figure 3). For this purpose, a new column named “matchContour” was added to the datasets and the code was designed to interpolate the geographical location of occurrences, with matching seafloor geographical areas of the shallow and deep datasets, accordingly. For the first case, occurrences that did not have depth recorded but were geographically located in each of these areas were included in the groups “*matchShallow*” and “*matchDeep*”, respectively. For the second case, the group “*shallowFromDeepSet*” was created to represent the occurrences that were recorded deeper than 500 m but lay geographically in the 0–500 m zone. This means that they would not belong to the deep dataset if coordinates are correct and follow the datum WGS84 (EPSG Geodetic Parameter Dataset 4326). The occurrences that lay in this group are disregarded from this point, as they may be misplaced in depth, coordinates, or both. Besides, the occurrences with matching depths and coordinates with the depth and geographical position of the shallow and deep datasets were added in the groups “*matchShallow*” and “*matchDeep*”, accordingly.

The second analysis is called raster analysis. This is used to process occurrences with depth into planktonic and benthic sets. We used bathymetric raster data for the AOI, contained in *.tiff* files, retrieved from the EMODnet server in April 2025 (Krystalli et al. 2023) and the GEBCO gridded bathymetric data (GEBCO Compilation Group 2024). The depth of each occurrence was contrasted with the bathymetry stored in the pixel matching the geographical position of each occurrence. Occurrences were classified into three groups, in a new dataset column named “tiffmatch”: “*tiffPlankton*”, “*tiffBenthos*” and “*tiffBelowDepth*”. Occurrences in the first and second groups encompassed the planktonic and the benthic sub‐datasets, respectively. Occurrences in “*tiffBelowDepth*” were disregarded from this point, as their recorded depth fell below the seafloor depth, thus not suitable for being classified either as planktonic or benthic occurrences. It is relevant to mention that geographical coverage of raster data between the EMODnet database (Krystalli et al. 2023), the GEBCO grid 2024 (GEBCO Compilation Group 2024) and the geographical position of occurrences did not exactly match in all cases. Consequently, some marine species occurrences lying along and close to the coast (4072) were excluded from raster analysis, as they did not hold an equivalent in raster data, despite having depth information. Resolution for data in this study corresponds to a 15 arc‐second interval grid (500 m per 500 m pixel size at the equator, approximately) for GEBCO data and of 1/16*1/16 arc min (115 m grid approximately) for EMODnet data.

#### 2.2.4 Biological Diversity

Before starting, any duplicated occurrences were checked and removed (902,504), comparing the columns *decimalLatitude, decimalLongitude, depth, day, month, year*, and *scientificName*. Similarly, all remaining datapoints lying outside the AOI and below the *Molloydypet* were removed (i.e., geographical and depth outliers). Species were quantified per latitudinal segments and depth zones and information on species richness, occurrences, and abundances was obtained for the shallow and deep datasets, for each of their 12 depth zones (i.e., both the shallow and deep zones). Species abundances were calculated by multiplying the number of occurrences by their row‐corresponding “individualCount” dataset column, then adding up the results for the same species name. The corresponding scripts in GitHub are scripts IVa and IVb.

#### 2.2.5 Spatial Analysis

Data was divided in the above mentioned datasets and exported to ArcGIS to count occurrences in map grid cells, following the methodology and criteria described by Troia and McManamay (2016). The AOI was divided into a grid of 122,955 cells of 0.1° by 0.1°, corresponding to 11.13 × 6.24 km (69.45 km^2^) at 56° N and 11.17 × 0.96 km (10.72 km^2^) at 85° N, as indicated in ArcGIS with the “measure” tool. Using the spatial join tool in ArcGIS, we allocated each occurrence in a corresponding cell located in their same geographical position; then data was exported to R. The range for the number of occurrences per grid cell was designated with logarithmic categories from A to F. Category A corresponds to 1–10, category B to 10^1^–10^2^, category C to 10^2^–10^3^, category D to 10^3^–10^4^, category E to 10^4^–10^5^, and category F to 10^5^–10^6^ occurrences. The “number of cells” represents how many grid cells, containing a given category, are present for a certain decade (e.g., 35 cell grids containing 1–10 occurrences each). For example, a decade (or the entire AOI) may contain several categories, each with a number of cells. The corresponding script in GitHub is script V.

## 3 Results

### 3.1 Occurrences and Coverage in the AOI and the Deep‐Sea Mining Opening Area

The AOI encompasses 10,505,496 marine species occurrences, which were filtered from 287,805,496 occurrences in source data, passing through the occurrence analysis (Table 2). About half of the occurrences are reported with depth (5,116,901) and around a quarter (2,534,968) had coincident raster information of depth to be classified as benthic or planktonic. From marine occurrences data with depth, 14,027 occurrences were classified as “*shallowFromDeepSet*” and 2,563,834 occurrences as “*tiffBelowDepth*”. For uncertainty information, 4,127,843 occurrences contained information about coordinate uncertainty and 6,939,230 and 213,704 occurrences had depth accuracies of ≤ ±100 m in the shallow and deep datasets, respectively. For data from GBIF and OBIS, 1,403,008 occurrences had NA or 0 values in the column *individualCount*.

**TABLE 2:** Number of occurrences for quality assessment of open‐source biodiversity data. (a) Outcomes from filters applied to obtain marine data. (b) Outcomes from the depth analysis (depth‐contouring and raster analysis).

(a)	Source data		Occurrence analysis			
			Land‐masking		Filter with WoRMS	
Decade	GBIF	OBIS	GBIF	OBIS	GBIF	OBIS
1876–1899	657,220	5568	41,693	1465	6072	1399
1900–1909	438,447	119,002	30,545	46,482	6740	21,520
1910–1919	547,804	23,758	35,991	6570	4523	3449
1920–1929	674,113	14,085	49,463	5819	9057	5256
1930–1939	1,218,308	12,843	83,596	6674	17,541	6615
1940–1949	910,748	29,401	34,780	8292	4016	5229
1950–1959	2,051,845	156,816	149,931	88,847	23,838	60,587
1960–1969	3,497,099	179,512	218,104	62,974	39,670	34,210
1970–1979	7,755,296	932,925	571,504	314,824	137,954	208,336
1980–1989	15,723,571	1,984,858	1,105,999	412,200	469,723	377,147
1990–1999	22,848,418	3,170,324	1,642,409	1,147,722	836,193	1,044,185
2000–2009	47,596,749	3,795,931	2,646,313	1,525,185	1,231,024	1,347,419
2010–2019	98,268,884	3,976,879	6,575,250	1,254,188	2,487,327	1,054,297
2020–2025	70,250,283	751,859	4,591,405	298,618	1,626,445	250,818
Semitotal	272,438,785	15,153,761	17,776,983	5,179,860	6,900,123	4,420,467
EMODnet		207,698		109,485		85,982
NNAexp		5252		3589		1428
Total		287,805,496		23,069,917		11,408,000
% (compared to source data)		100		8.1		3.9
			Total after occurrence analysis (with duplicates)			11,408,000
			Occurrences with depth (no duplicates)			5,116,901
			Occurrences without depth (no duplicates)			5,388,595
			Geographical outliers (excluded)			136
			Depth outliers (excluded)			244
			Total occurrences without duplicates			10,505,496

(b)	Depth‐contouring analysis		Raster analysis: planktonic and benthic			
Interval	Shallow	Deep	Shallow planktonic	Shallow benthic	Deep planktonic	Deep benthic
1876–1899	5376	2871	1198	163	529	33
1900–1909	19,164	5725	1382	163	82	1
1910–1919	5056	1091	41	11	8	0
1920–1929	12,733	1329	712	148	6	0
1930–1939	21,000	2906	1123	1028	45	103
1940–1949	7655	894	1590	32	1	0
1950–1959	74,674	3062	45,446	6330	165	16
1960–1969	60,574	2001	30,186	247	99	22
1970–1979	191,867	3044	36,048	371	747	64
1980–1989	601,000	35,446	87,923	1109	312	83
1990–1999	1,537,379	98,333	512,140	8011	7037	138
2000–2009	2,464,239	65,126	835,886	19,077	22,474	943
2010–2019	3,413,371	61,665	689,256	48,480	29,737	3243
2020–2025	1,780,235	27,680	101,882	28,199	9882	1016
	10,194,323	311,173	2,344,813	113,369	71,124	5662
Total, without duplicates		10,505,496	Total matching raster data			2,534,968
*shallowFromDeepSet* (excluded)		14,027	Total planktonic			2,415,937
*tiffBelowDepth* (excluded for raster analysis)		2,563,834	Total benthic			119,031

*Note:* The last decade covers data from 2020 to the present time.

Abbreviation: NNAexp, Norwegian North Atlantic Expedition 1876‐1878.

#### 3.1.1 Deep and Shallow Datasets

**FIGURE 4:**
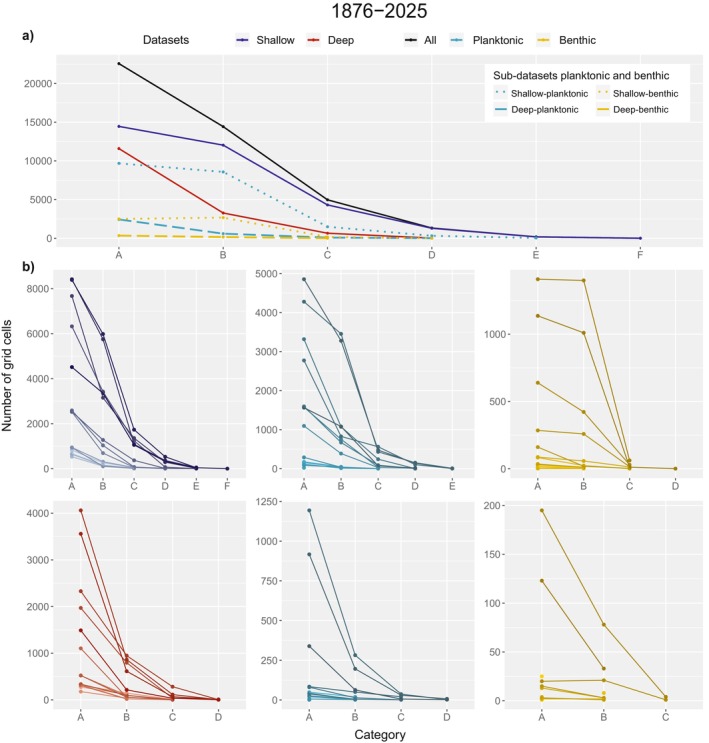
Number of 0.1° × 0.1° map grid cells (out of 122,955 in total), with marine species occurrences for the shallow (blue) and deep (red) main datasets and the planktonic (cyan) and benthic (gold) sub‐datasets. (a) For all data in the time series. (b) For each main dataset and their corresponding sub‐datasets. The number of occurrences per grid cell has categories from A to E in a logarithmic scale. A: 1–10 occurrences, B: 10^1^–10^2^ occurrences, C: 10^2^–10^3^ occurrences, D: 10^3^–10^4^ occurrences, E: 10^4^–10^5^ occurrences, F: 10^5^–10^6^ occurrences. In (a), shallow and deep occurrences of the planktonic (cyan) and benthic (gold) sub‐datasets are represented by dotted and dashed lines, respectively. Note that these sub‐datasets include occurrences with depth records only and then, have much lower values when compared to the shallow and deep main datasets (which include occurrences with and without depth recorded). The black line corresponds to the full dataset in this work (all data, 10,505,496 occurrences). In (b), lighter tones represent older decades and darker tones, most recent decades in the time series.

From the total of marine occurrences, 10,194,323 of them belong to the shallow dataset (97%) and 311,173 to the deep dataset (3%). For the opening area, the number of occurrences was 8300 and 10,027, respectively. For 122,955 map grid cells in the AOI (i.e., 100%), 43,470 include datapoints (35%) (Figure 4a); 32,274 contain data from the shallow dataset, with categories ranging from A to F, and 15,528 grid cells encompass data from the deep dataset, with categories ranging from A to D (Figure 4). For both the shallow and deep datasets, A is the category with the most grid cell counts (14,456 and 11,589, respectively). Additionally, only the shallow dataset contains grid cells in the E‐category (183), from which 40 are planktonic, thus identified with depth, and 4 F‐category grid cells (Figure 4b), located along the south coast of Norway. For individual decades, the shallow dataset has the highest A‐category number of grid cells from 2000 to 2009 (8421 grid cells) and the deep dataset from 2000 to 2009 (4061 grid cells) (Figure 4b).

#### 3.1.2 Benthic and Planktonic Sub‐Datasets

From marine occurrences, planktonic data accounts for 2,415,937 occurrences (23%) and benthic data for 119,031 occurrences (1%). From the 43,470 grid cells with data from the spatial analysis, 20,098 contain data from the shallow‐planktonic dataset, with categories ranging from A to E; 5329 grid cells encompass data from the shallow‐benthic dataset, with categories ranging from A to D; 3127 have data from the deep‐planktonic dataset, with categories ranging from A to D and 507 grid cells encompass data from the deep‐benthic dataset, with categories ranging from A to C (Figure 4). For planktonic and benthic sub‐datasets, A‐category grid cells are also the most recurrent: shallow‐planktonic (9680); deep‐planktonic (2433); shallow‐benthic (2498) and deep‐benthic (344). These results may differ if applying the raster analysis with a more detailed resolution capacity, as these sub‐datasets are estimated through a raster geographical equivalent in depth.

**FIGURE 5:**
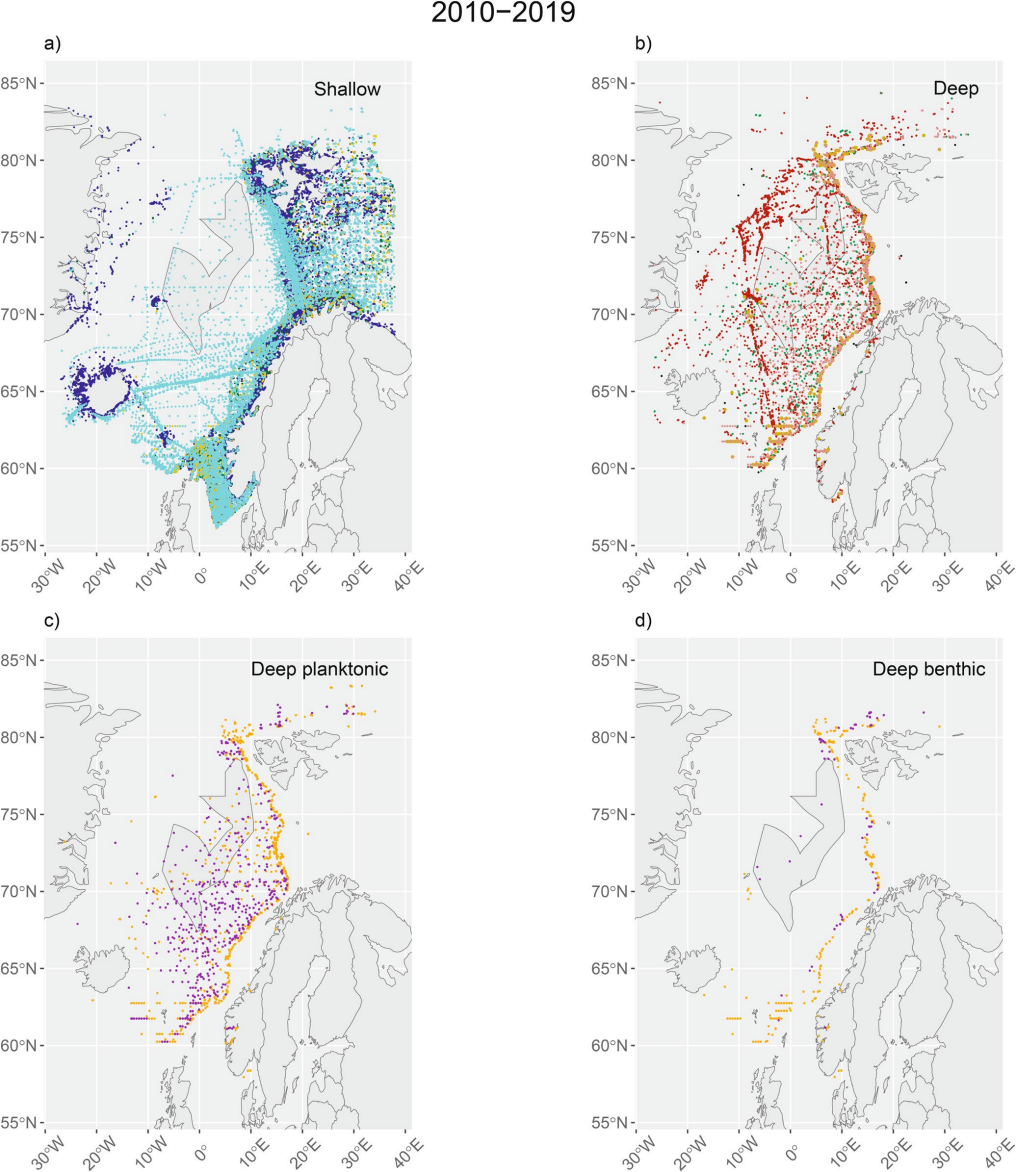
Shallow (a), deep (b), deep‐planktonic (c), and deep‐benthic (d) datasets in map view. Gold data points in (b) are plotted with bigger dots to facilitate reading. Blue = *matchShallow*, red = *matchDeep*. For (a) and (b): Pale tone = *tiffPlankton*, dark tone = occurrences without depth, gold = *tiffBenthos*. Dark green = *tiffBelowDepth*, black = *shallowFromDeepSet*. For (c) and (d), Orange = mesopelagic/sublittoral, violet = bathypelagic/bathyal, green = abyssopelagic/abyssal. For other decades in the time series, see Appendix (Figures A1, A2, A3, A4, complementary map views). Note that planktonic and benthic data include occurrences reported with depth.

**FIGURE A1:**
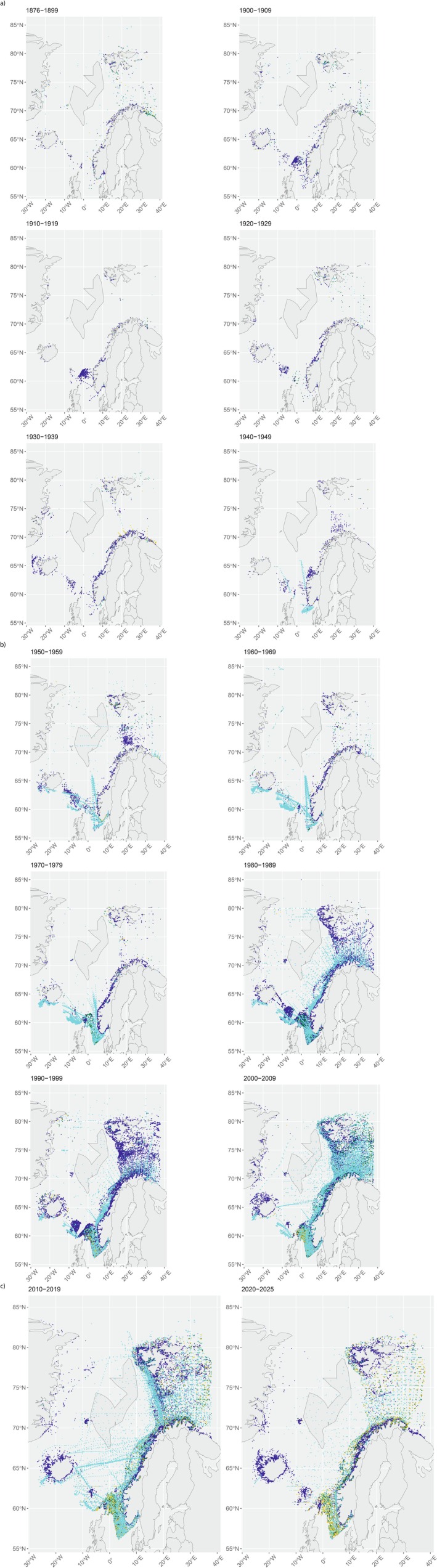
Shallow dataset in map view. (a) 1876–1949; (b) 1950–2009 and (c) 2010–2025.

**FIGURE A2:**
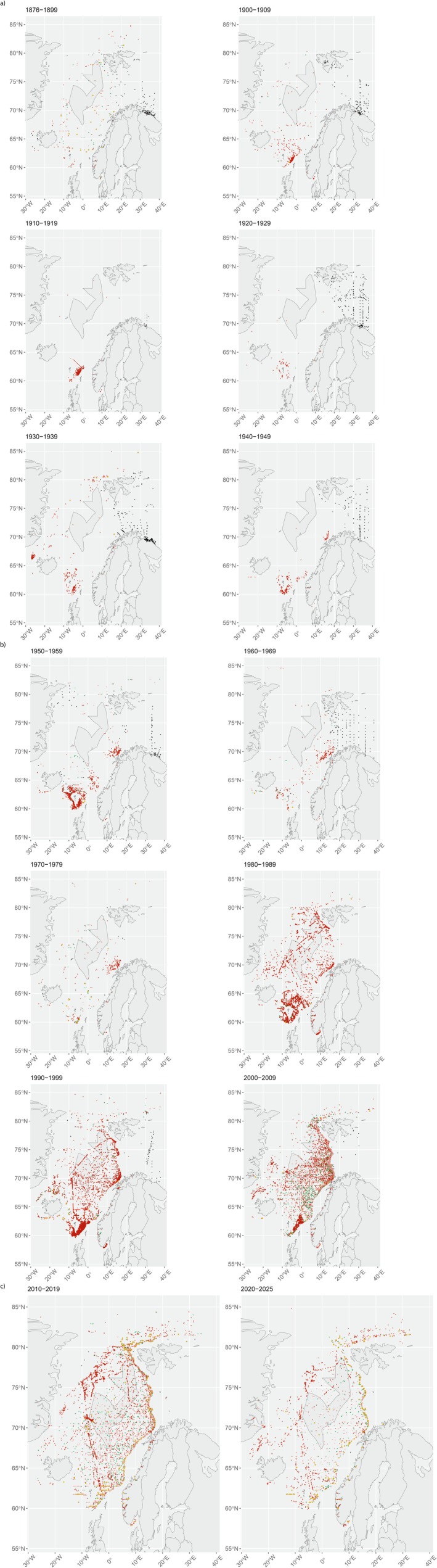
Deep dataset in map view. (a) 1876–1949; (b) 1950–2009 and (c) 2010–2025.

**FIGURE A3:**
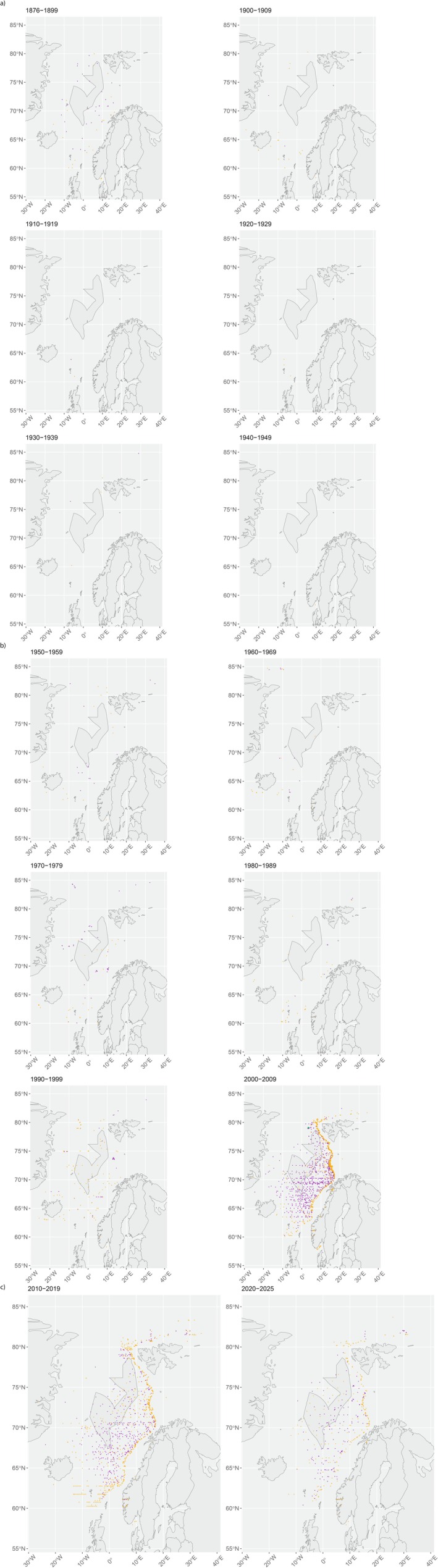
Deep‐planktonic dataset in map view. (a) 1876–1949; (b) 1950–2009 and (c) 2010–2025.

**FIGURE A4:**
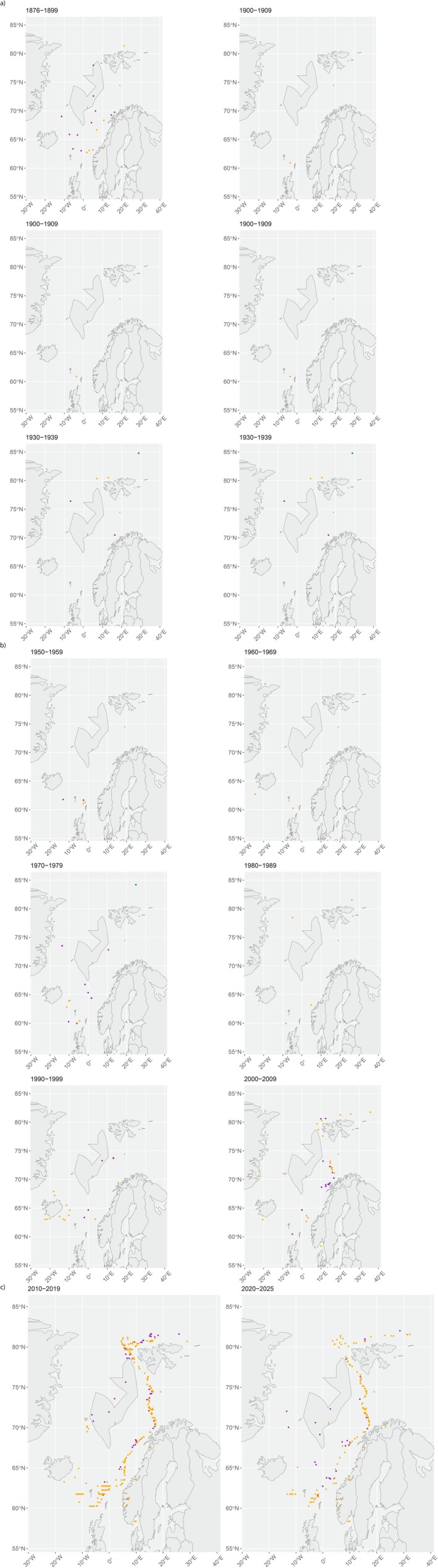
Deep‐benthic dataset in map view. (a) 1876–1949; (b) 1950–2009 and (c) 2010–2025.

In map view for the AOI, most occurrences from the total are planktonic and belong to the shallow dataset (Figure 5a,b) in the whole time series (see Appendix, Figures A1, A2, A3, A4, complementary map views). Most of them are present in southern latitudes, near the coast and in the Barents Sea. Occurrences without depth are more prevalent in the west than in the east of the AOI. For data with depth in the deep dataset, most occurrences are planktonic and recorded in the mesopelagic area (Figure 5c) and most benthic occurrences are located along the continental shelf break, in the sublittoral‐bathyal area (Figure 5d). Occurrences with matching raster data are distributed mainly in the epipelagic/sublittoral province (1,591,876 occurrences, ~63%), followed by the mesopelagic/sublittoral province (916,262 occurrences, ~36%), the bathypelagic/bathyal province (26,788 occurrences, ~1%) and the abyssopelagic/abyssal province (42 occurrences, ~< 1%).

### 3.2 Biodiversity Over Time

**FIGURE 6:**
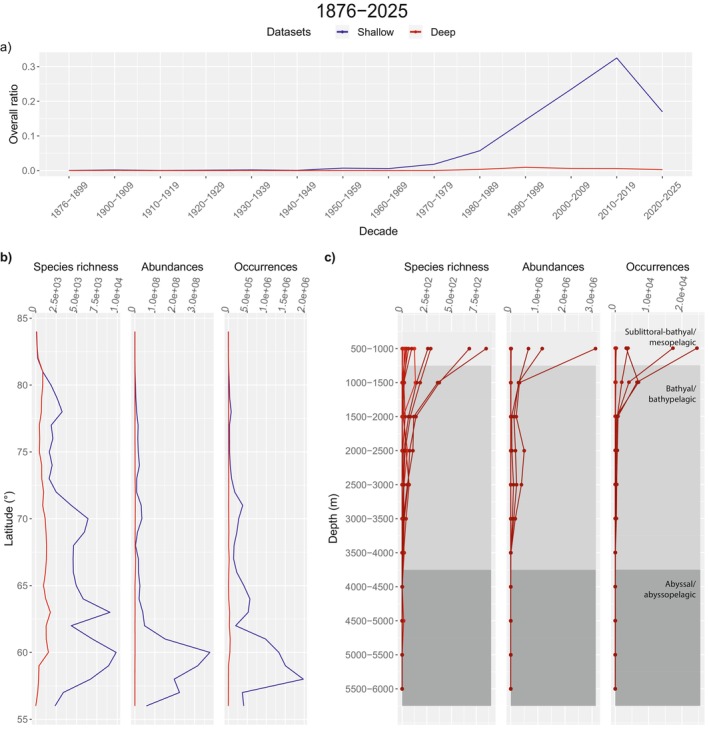
Biodiversity over time, latitudes and depth zones. (a) Overall ratio of marine occurrences for each decade. This ratio corresponds to the number of occurrences in a certain decade over all occurrences (in both shallow and deep datasets). (b) Species richness (number of species), abundances (number of individuals, see methods, Section 2.2.4) and occurrences (number of individual occurrences) for the whole region, examined along latitudinal segments, and (c) depth zones each 500 m in the deep dataset. In (c), regions in gray represent the boundaries between oceanic provinces in the deep dataset (sublittoral‐bathyal/mesopelagic; bathyal/bathypelagic; abyssal/abyssopelagic) and different lines in red, the various decades in this dataset. Blue lines indicate the shallow dataset and red lines, the deep dataset.

For species richness, abundances and occurrences in the whole time series, the shallow dataset presents a peak for occurrences between 2010 and 2019 and the deep dataset between 1990 and 1999 (Figure 6a). This peak rises mainly from 1970 to 1979 for the first and from 1980 to 1989 for the second. The maximum number of species at a given latitude (from west to east), for the shallow and deep datasets, is for 60° N (approximate latitude for Bergen) and 63° N (approximate latitude for Trondheim), with 9863 and 1792 marine species, respectively, reported to the time of data collection for the whole AOI (Figure 6b). For the deep dataset, values of species richness are higher for shallower depth zones, especially for the 500–1000 m depth zone. Species richness decreases to < 200 species in all decades, from deeper than 1500 m and occurrences values fall abruptly from that depth. Abundances peak at two depth zones, besides the shallowest zones: 2000 m to 2500 m and 2500 m to 3000 m (Figure 6c).

**FIGURE 7:**
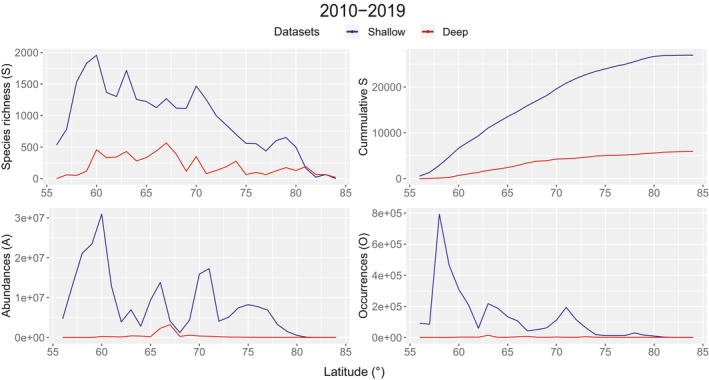
Species richness, accumulation curve, occurrences and abundances along latitudinal segments for the decade 2010–2019 (see Appendix, Figure A5, A6, A7, complementary plots, for the entire time series). Blue lines correspond to the shallow dataset and red lines, to the deep dataset. Accumulation curves may represent very early stages for the filling of the species pool, instead of describing the plateau reached when the pool is complete (see Section 4).

**FIGURE A5:**
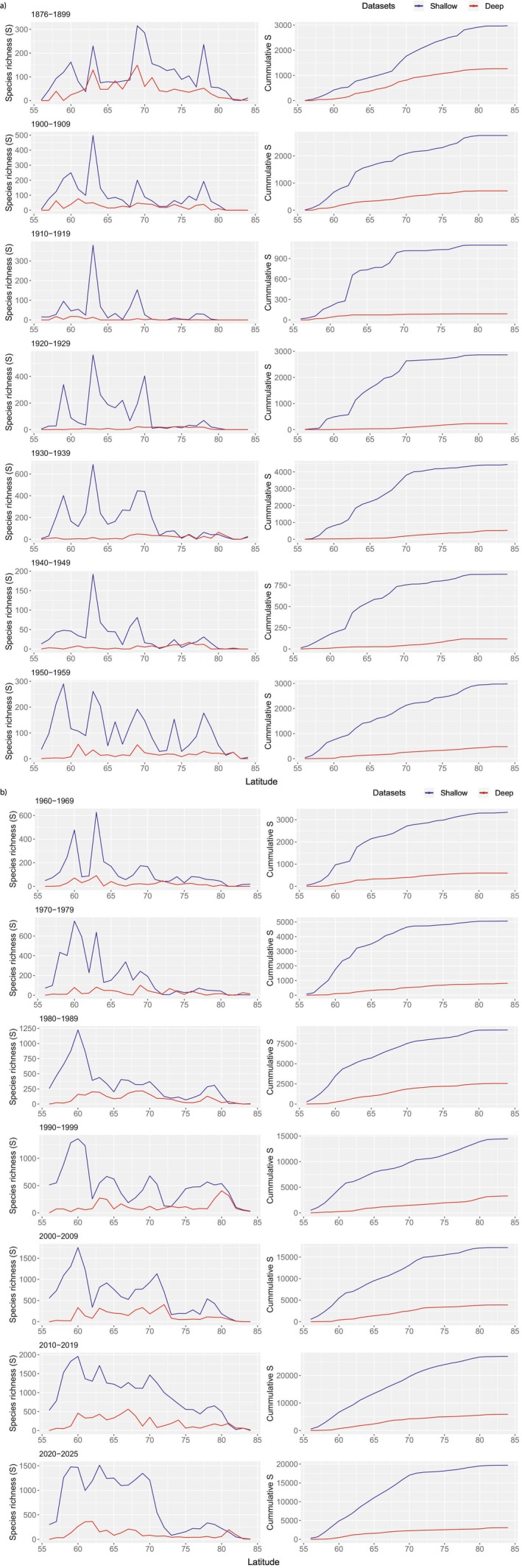
Species richness and accumulation curves along latitudinal segments. (a) 1876–1959 and (b) 1960–2025.

**FIGURE A6:**
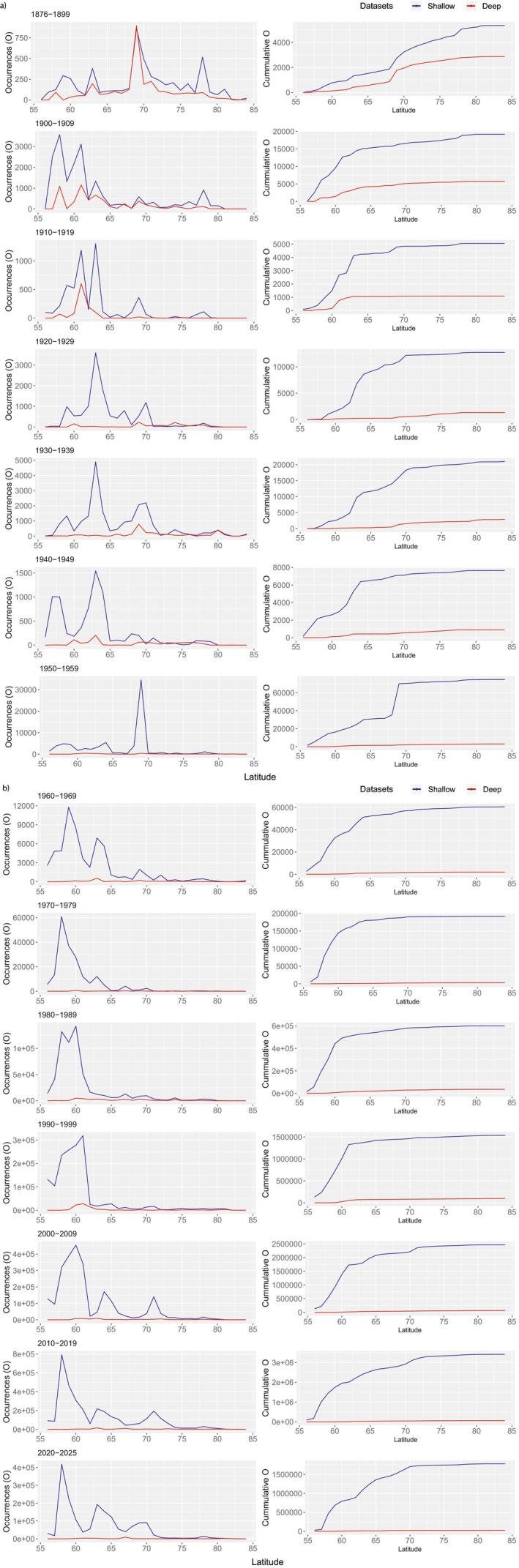
Occurrences and accumulation curves along latitudinal segments. (a) 1876–1959 and (b) 1960–2025.

**FIGURE A7:**
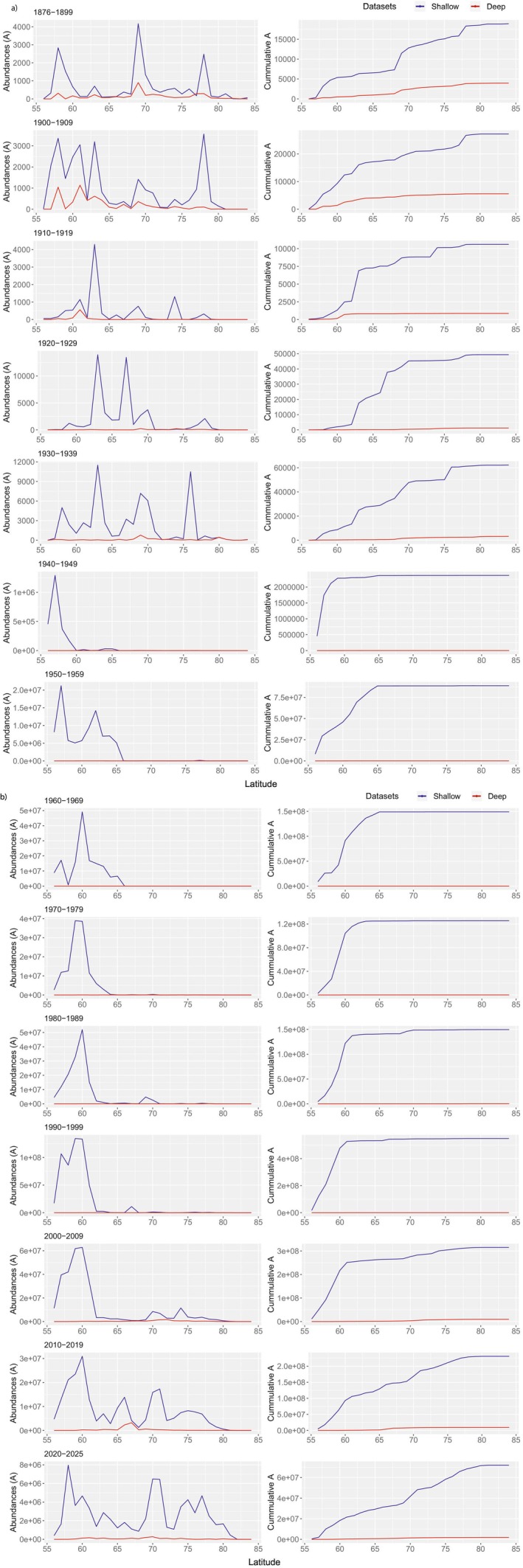
Abundances and accumulation curves along latitudinal segments. (a) 1876–1959 and (b) 1960–2025.

Particularly for decades, 2010 to 2019 has the highest report of species richness, with 1957 species (60° N) for the shallow dataset and 565 species (67° N) for the deep dataset (Figure 7). In the same decade, for instance, the maximum value in the cumulative curve of species richness accounts for 26,945 species for the shallow dataset and 5910 for the deep dataset. A pattern of direct proportionality between the number of occurrences and the cumulative curve of species richness is constant along the whole time series (see Appendix, Figures A5, A6, A7, complementary plots for all decades). To the north, species richness values for the deep dataset surpass species richness values for the shallow dataset. Peaks in abundances seem to be accentuated by the influence of individual counts.

## 4 Discussion

Occurrences of marine species in the AOI are concentrated in the shallow and planktonic areas (Figures 4 and 5) and spatial coverage of data is still scarce for the deep dataset, as there are occurrences of this set in 35% of the grid cells of the AOI. In the shallow dataset, the 32,274 grid cells contain mainly 1–10 occurrences (i.e., category A) and for the deep dataset specifically, the number of grid cells falls to 15,528, predominantly in the category A (Figure 4b). Thus, approximately the half of map grid cells with data in the AOI correspond to the category A. Therefore, it is difficult to identify a consistent pattern of biodiversity change every 10 years, but it is possible to quantify biodiversity knowledge gaps and establish general patterns, as this will aid in identifying specific‐case studies as strategic localities for sampling of deep‐sea fauna.

Taxonomical accuracy is important for understanding how occurrences decrease from source data throughout quality control, to then be integrated into the four datasets (i.e., shallow‐planktonic; shallow‐benthic; deep‐planktonic and deep‐benthic) (Figure 2). About half of the records after the occurrence analysis (and therefore, after the land‐masking process) are dropped (Table 2), therefore taxonomical verification is central to processing of data. The World Register of Marine Species (WoRMS) is an important source for comparing information about marine species, helping to understand and distinguish challenges related to species identification. In general, undescribed marine species have reached a proportion of what would be two‐thirds of the total marine species for the beginning of this century (Appeltans et al. 2012) and in 2017, only 16% of all named species were marine (Costello and Chaudhary 2017). WoRMS becomes, in this sense, a highly relevant taxonomic tool to provide precision in species estimates by the recognition of valid names. An accepted name might be recorded yet as a non‐accepted name (usually old genus and/or species names) in scientific databases. These non‐accepted names might also be included in scientific papers and could be widely used, even being listed alongside the accepted names. Tailored algorithms try to reconcile these taxonomical differences (e.g., Rees 2014) and multiple suggestions have been proposed to curate data (e.g., Hackett et al. 2019; Urbano et al. 2023; Veiga et al. 2017), under the principles of FAIR (findability, accessibility, interoperability and reusability) in data sharing (Urbano et al. 2023). Taxonomical determination is therefore essential to the identification of marine biodiversity, then deep‐sea biodiversity yet to be discovered.

Geographical and depth accuracy precisions need to be considered as well. For excluded occurrences from the groups “*shallowFromDeepSet*” and “*tiffBelowDepth*” (Figure 5, see materials and methods), information in the first might also be part of the shallow dataset, as those occurrences seem to come from surveys between the north coast of Norway and Svalbard, mainly from before 1970. The second group is largely located east of the continental shelf break and seem to lie mainly on the eastern part of the AOI. Coordinate accuracy ‐denoted by the database column “*coordinateUncertaintyInMeters*”‐ is recorded for only 39% from the total of marine occurrences (4,127,843). This coincides with information from Marcer et al. (2021), who noted that of the 10% of the scientific databases accessible digitally worldwide, only 31% have coordinate uncertainty information for both terrestrial and marine occurrences. Additionally, there is a considerable number of occurrences without depth information (Table 2) and many of them are flagged as “NO_DEPTH”. This is congruent with the category *human observations*, described in the “*basisOfRecord*” database column from source data in source databases. Sufficient metadata attached to individual datasets in data aggregation services like GBIF is then crucial due to its role in helping understand the collection protocols and usefulness of individual datasets (Heberling et al. 2021), hence holding spatial–temporal information about marine biodiversity (Proença et al. 2017).

Possible sampling biases were treated with care in data analysis, especially considering lower sampling effort of Arctic and benthic fauna and representativity of data for the calculation of abundance values (Figures 5, 6b and 7). Both for GBIF and OBIS, 13% of occurrences were reported with either NA or 0 values for the column *individualCount*, and individual counts may be further accentuated by the method that was used for sampling. From the Norwegian North Atlantic Expedition, for example, living and dead specimens were collected relying on the type of sampling conditions at the time. Different methods of sampling may have been used for old and new measurements, such as sampling with nets or bottom trawling (e.g., Jacquemont et al. 2024; Mohn et al. 1880), which may bias the number of individuals counted for specific groups. In consequence, individual counts seem to accentuate the highest values for occurrences, but they may obscure the rarest ones. This may be reflected in the number of planktonic occurrences compared to benthic occurrences, when the sampling method are nets, for example. For benthic fauna, biodiversity measures are related to the type of substrate which allows to infer relationships between the organisms (e.g., predator–prey), substrate (i.e., hard vs. softbottom) and ecosystem resources present (e.g., preys in a trophic structure) (Finke and Denno 2005; Jumars et al. 2015). Ecological functions as well as the identification of species roles (Brodie et al. 2018) in the regional species pool are related to species identities, becoming even more relevant for an ecosystem such as the Arctic Ocean at the north of the AOI, for instance. This system encompasses complex climatic and oceanographic interactions due to the dynamics of its ice cover (Kayode‐Edwards et al. 2024).

Recognition of these biases enables a better understanding of results. When comparing occurrences in the shallow and deep datasets, the deep dataset possesses considerably less occurrences than the shallow dataset (Figure 7) for the whole time series (see Appendix, Figure A5, A6, A7, complementary plots). This reduction is evident when mapping occurrences (Figures 5 and 6) and may be explained by the presence of shallow occurrences in the Barents Sea, east of the AOI, a shelf sea with a mean depth of 230 m, therefore located in the shallow area of the extended continental shelf. For areas other than this sea (i.e., North Sea, Norwegian Sea and Greenland Sea), most occurrences are shallow and planktonic similarly. This seems influenced by shallow sampling (i.e., < 500 m for this work) in transects, from shallow coastal to deep‐sea areas, resembling the shape of vessel trajectories, which are traced by contiguous points in linear shapes in map view (Figure 5a). For the deep dataset and visibly after 2009, most of the occurrences are planktonic (Appendix, Figure A2, complementary map views). This is consistent with a higher precision of measurements for depth (i.e., “*depthAccuracy*”) in the shallow dataset when compared to the deep dataset (6,939,230 occurrences vs. 213,704 occurrences with depth accuracies of ≤ ±100 m). As the opening area encompasses national jurisdiction as defined by the extended continental shelf (Ministry of Foreign Affairs 2020), the efforts for environmental planning in these areas may be actioned jointly with national measures for ecosystem management, such as Other Effective Area‐Based Conservation Measures (OECM) (Gurney et al. 2021; Maxwell et al. 2020). This may cover Vulnerable Marine Ecosystems (VME), which have been advised to be priority for mapping (e.g., Morato et al. 2021). Increasing species occurrence data and its precision may count towards the number of occurrences used for biodiversity assessments, based on open‐source data and especially for the deep benthic area. This is also reflected in the number of occurrences inside the assessment area/proposed area/opening area for deep‐sea mining (Figure 5).

Following results, peaks of occurrences and species richness may indicate knowledge gaps on rare species. Peaks in occurrence values tend to coincide with peaks in species richness values along latitudes for both the shallow and deep datasets for all the time series (Figure 7 and Appendix, Figures A5, A6, A7, complementary plots). Even if peaks for occurrences have lower values in northern latitudes than in southern ones, latitude‐corresponding species richness peaks are still present along the AOI (Figure 6b). Particularly, for the northernmost latitudes of the AOI, it is possible to see a peak in species richness in the deep dataset, which tends to be more pronounced than the one in the shallow dataset (Figure 6a). Besides, when observing depth zones every 500 m in the deep dataset, occurrences contribute to records of species. Data is consistent, for instance, with a reported lack of benthic biodiversity data for the Arctic, north of 66° N, when including information from GBIF and OBIS (Ramirez‐Llodra et al. 2024). This is contrasting to the southern part of the AOI, as the North Sea is considered a well‐studied and the most sampled area. However this last also includes an unknown range of cryptic species. Rare species and/or scarcely sampled species (e.g., Ramirez‐Llodra et al. 2020) may reveal bathymetric gradients in the Norwegian Sea, as suggested by habitat shifts in gastropods and polychaetes (Høisæter 2010; Oug et al. 2017). For example, changes in water masses and their temperature have been suggested as explaining factors in habitat shifts and distribution due to stable sub‐zero temperatures in water masses deeper than 600–800 m in the Nordic Seas (Høisæter 2010; Oug et al. 2017). This information can be especially important for data of benthic fauna sampled along the Mid Oceanic Ridge (e.g., Eilertsen et al. 2024).

Temporal and spatial knowledge gaps found, including those depth‐related, could hinder evidence‐based decision‐making for environmental impact assessments. Moreover, there is not enough data to confidently catalogue species identities in the full species pool of the AOI. Such a catalogue would provide a basis to calculate area‐based ecological indices from absence and presence species counts and therefore, perform biodiversity‐related estimations. The area encompassed by the AOI depicts a regional scale, thus, a corresponding species pool. In this sense, a single grid cell area would represent a measure close to a local scale (10.61–69.45 km^2^) inside a regional area such as the AOI (7.3 × 10^6^ km^2^) (Figure 1). When a species pool is filled, the slope of the species accumulation curve reaches a plateau after a second inflection point (i.e., a constant asymptote) (e.g., Thompson et al. 2003). However, this asymptote is not evident, indicating a lack of species richness data to complete the species pool in all the time series (Figure 7 and Appendix, Figure A5, A6, A7, complementary plots). For the shallow dataset, the curve is still being actively filled, as evidenced by a consistent slope in the accumulation curve along latitudes. The quick increase of the slope of the accumulation curve, often pictured as a linear slope following regular sampling, has not yet been reached for the deep dataset. A strong relationship between occurrences curves and species accumulation curves is consistent along all the time series (see Appendix, Figures A5, A6, A7, complementary plots), showing that the slope in the curve decreases abruptly due to lack of sampling. Therefore, these findings suggest coherence with multiple authors stating that the deep sea is undersampled and that more studies in this ecosystem are highly advisable (e.g., Grassle 1989; Schiaparelli et al. 2016; Hughes et al. 2021).

When speaking about the deep dataset in particular, possible patterns of species distribution can be observed as a starting point. In the AOI, most species richness lies on the top 2000 m, along the 500‐m depth zones for this dataset (Figure 6c). One coinciding pattern corresponds to the distribution of polychaetous annelids as occurrence values peak in the 500–1000 m depth range (Figure 6c) and abundance values present the same pattern. The study of the annelids was based on large samples from 1981 to 1987 from 500 m and deeper (Oug et al. 2017) and found that most species live in the upper and middle continental slope (500‐1000 m). Another peak in species richness is found in the zone of 2500–3000 m (Figure 6c), which coincides with a peak in occurrences. The results on polychaete distribution (Oug et al. 2017) corroborated similar results on gastropods (Høisæter 2010), partly based on the same samples, which are currently not available in public databases. The Norwegian Sea and the southern part of the Barents Sea have been mapped by the MAREANO programme (Buhl‐Mortensen et al. 2015) to evaluate anthropogenic impacts (i.e., bottom trawling and litter), especially near the coast and in the sublittoral and bathyal zones (Buhl‐Mortensen and Buhl‐Mortensen 2018). As sampling information is extensively concentrated in coastal and shallow areas, this largely counts for measures of marine biodiversity (i.e., species richness) when contrasted to deep‐sea data (Figure 6b). Therefore, deep‐sea biodiversity patterns need close attention, especially in the context of deep‐sea mining activities.

In conclusion, as horizons derived from this work, habitats holding the ore for mining, such as hydrothermal vents, or the slope of seamounts holding manganese‐rich crusts in the AOI, should be a priority for research, giving special attention to benthic fauna living in the substrate. Data quality in publicly available sources and reported as species occurrences, has a limited extent to knowledge of biodiversity in the region assessed here, regarding the actual number of occurrences available for analyses. All these quantitative challenges reflect the need for better accuracy and resolution in taxonomic and depth data for mapping of marine species, therefore the assessment of degradation of their ecosystem by deep‐sea mining (e.g., Wing‐Gabrielsen 2025). Ecosystems affected can include cold‐water coral reefs, sea pen communities, cold‐water coral gardens, deep‐sea sponge aggregations and active hydrothermal vent fields (Kazanidis et al. 2020; Morato et al. 2021) and others, for example, which can be explorable by Remote Operated Vehicles (ROV), for instance (Johnsen et al. 2016; Ludvigsen et al. 2018). Video systems, scientific benthic sampling, and expert judgment may also provide an actionable way to collect this information (e.g., Morato et al. 2021). Finally, considering published papers on taxonomic groups, a broader range of occurrences and taxonomic resolution will be available once they are shared publicly as open‐source data.

## Author Contributions

**Laura C. Paiba‐García:** conceptualization (lead), data curation (lead), formal analysis (lead), investigation (lead), methodology (lead), project administration (supporting), software (lead), validation (equal), visualization (lead), writing – original draft (lead), writing – review and editing (lead). **Geir Johnsen:** conceptualization (lead), data curation (supporting), formal analysis (equal), investigation (equal), methodology (equal), project administration (equal), supervision (lead), validation (equal), visualization (supporting), writing – original draft (equal), writing – review and editing (equal). **Sam Wenaas Perrin:** data curation (supporting), methodology (supporting), software (supporting), writing – review and editing (supporting). **Torkild Bakken:** conceptualization (lead), data curation (lead), formal analysis (equal), investigation (equal), methodology (equal), project administration (lead), software (supporting), supervision (lead), validation (equal), visualization (supporting), writing – original draft (equal), writing – review and editing (equal).

## Conflicts of Interest

The authors declare no conflicts of interest.

## Data Availability

The code that supports the findings of this study is openly available in GitHub repository at https://github.com/PhDprojectBio/Marine‐biodiversity‐in‐the‐Norwegian‐deep‐sea‐mining‐opening‐area.

## Appendix A

This section includes figures and graphics for the entire time series.

### Complementary Map Views

Gold data points in Figure A2, green data points in Figure A3, and all data points in Figure A4 are plotted with bigger dots to facilitate reading. Figures contain the opening area plotted in the area corresponding to the Norwegian Sea.

- Figure A1: Shallow dataset in map view. Blue = *matchShallow*. Pale tone = *tiffPlankton*, solid tone = occurrences without depth, gold = *tiffBenthos*. Dark green = *tiffBelowDepth*, black = *shallowFromDeepSet*.
- Figure A2: Deep dataset in map view. Red = *matchDeep*. Pale tone = *tiffPlankton*, solid tone = occurrences without depth, gold = *tiffBenthos*. Dark green = *tiffBelowDepth*, black = *shallowFromDeepSet*.
- Figure A3: Deep‐planktonic dataset in map view. Orange = mesopelagic/sublittoral, violet = bathypelagic/bathyal, green = abyssopelagic/abyssal.
- Figure A4: Deep‐benthic dataset in map view. Orange = mesopelagic/sublittoral, violet = bathypelagic/bathyal, green = abyssopelagic/abyssal.

### Complementary Plots

Blue lines correspond to the shallow dataset and red lines correspond to the deep dataset. The accumulation curves may represent very early stages for filling the species pool, instead of describing the plateau reached when the pool is complete (see Section 4).

- Figure A5: Species richness and accumulation curves along latitudinal segments.
- Figure A6: Occurrences and corresponding accumulation curves along latitudinal segments.
- Figure A7: Abundances and corresponding accumulation curves along latitudinal segments.

Link to the GitHub repository for this work, including all data scripts and relevant files for their use: Marine‐biodiversity‐in‐the‐Norwegian‐deep‐sea‐mining‐opening‐area.
